# EZH2 is increased in paediatric T-cell acute lymphoblastic leukemia and is a suitable molecular target in combination treatment approaches

**DOI:** 10.1186/s13046-015-0191-0

**Published:** 2015-08-14

**Authors:** V. D’Angelo, A. Iannotta, M. Ramaglia, A. Lombardi, M. R. Zarone, V. Desiderio, M. C. Affinita, G. Pecoraro, M. Di Martino, P. Indolfi, F. Casale, M. Caraglia

**Affiliations:** Department of Woman, Child and General and Specialized Surgery, Pediatric Oncology Unit - Second University of Naples, Via Luigi De Crecchio 4, 80138 Naples, Italy; Department of Biochemistry, Biophysics and General Pathology, Second University of Naples, Via S.M. Costantinopoli, 16, 80138 Naples, Italy; Department of Experimental Medicine, Section of Biotechnology and Medical Histology and Embryology, Second University of Naples, Naples, Italy

**Keywords:** EZH2, DZNep, T-cell Acute Lymphoblastic Leukemia

## Abstract

**Background:**

T-cell Acute Lymphoblastic Leukemia (ALL) represents about 10–15 % of pediatric ALL cases. EZH2, one of the components of Polycomb group proteins (PRC2) complex, catalyzes the trimethylation of histone H3 lysine 27 that is associated with transcriptional repression and tumor development.

**Methods:**

We examined the expression levels of PRC2 complex in primary samples of T cells ALL at diagnosis by western blotting and real time PCR. We evaluated the effect of 3-deazaneplanocin-A (DZNep), an EZH2 inhibitor, alone and in combination with Daunoblastine on cell viability, apoptotic death and cell cycle distribution of T cell established Jurkat cell line.

**Results:**

EZH2 was expressed in 75 % samples at different extents mainly with high expression level. SUZ12 was expressed in 60 % samples and EED in all samples, respectively. The Kaplan-Meier analysis shows that T-ALL expressing EZH2 had a lower probability of disease-free survival (DFS) compared to T-ALL negative for EZH2 (23 % vs 100 %) (p = 0.01). The EZH2 inhibitor DZNep used in combination with Daunoblastine was synergistic in inducing growth inhibition and increasing the apoptosis in T-ALL Jurkat cells at 48 and 72 h paralleled by EZH2 decreased expression. Moreover, the combination decreased the activity of Erk-1/2 proliferation enzymes with no effects on Akt survival pathway.

**Conclusions:**

The evaluation of EZH2 expression in pediatric T-ALL can be useful in predict the clinical outcome of the patients and EZH2 can be a useful target to improve the efficacy of conventional chemotherapy in this subset of patients with bad prognosis.

## Background

Epigenetic alterations have an important role in leukemogenesis and Polycomb group proteins, which consist of two complexes (PRC1 and PRC2), represent a major class of epigenetic regulators in development and transcriptional repression. The polycomb repressive complexes (PRC) are key mediators of transcriptional repression and consists of three core subunits: EED (embryonic ectoderm development), SUZ12 (suppressor of zeste 12 homolog) and EZH2 (enhancer of zeste homolog 2). PRC2 controls the pivotal methylation of lysine 27 of histone H3 (H3K27) catalyzed by the SET-domain containing enhancer of zest homolog 2 (EZH2) protein and its cofactors [1]. Deregulation of this histone modification can lead to epigenetic silencing of genes that promote differentiation and leads to leukemogenesis [2]. Components of PRC2 are required for embryonic development and notably loss of EZH2 gene is associated with a block in B- and T-cell differentiation. Moreover, EZH2 acts as an oncogene, as it is overexpressed in many solid cancers and lymphomas, in both advanced and metastatic diseases [3].

EZH2 is one of several chromatin regulatory proteins that have prompted great clinical and scientific interest as they offer the possibility of new therapeutic targets in cancer [4]. EZH2 contributes to cell fate decisions, orchestrating gene expression to control the balance between self-renewal and differentiation and has a predominant role in embryonic development becoming down regulated in some adult differentiated tissues [5].

T-cell acute lymphoblastic leukemia (T-ALL) represents about 10–15 % of pediatric ALL cases and is generally associated with unfavorable clinical features and aggressive biologic behavior such as higher risk for primary resistant disease, early relapse and isolated central nervous system (CNS) relapse compared to B-progenitor ALL patients. The prognosis of T-ALL in children and adolescents has improved in recent years as a result of more intensive chemotherapy approaches but it remains worse compared to B-lineage ALL especially in presence of poor initial response to prednisone therapy [5].

Recent articles showed that EZH2 knockdown results in a significant decrease in cellular proliferation and invasiveness, suggesting that epigenetic therapy targeting PcG machinery to treat various tumors, and the development of drugs inhibiting the trimethylation of the lysine 27 on histone 3 (H3K27me3) can be effective therapy [6]. H3K27 methylation is catalyzed by the SET domain of EZH2 and is helped by 2 additional proteins, embryonic ectoderm development (EED) and suppressor of zeste 12 (SUZ12). These proteins, together with the histone binding proteins retinoblastoma binding protein 4 (RBBP4) and RBBP7, form the core components of PRC2 [7]. Other proteins such as PHD finger protein 1 (PHF1), which specifically stimulates H3K27 trimethylation rather than dimethylation sirtuin 1 (SIRT1), and jumonji, AT rich interactive domain 2 (Jarid2) are also found in PRC2 complexes likely acting as modulators [8].

3-deazaneplanocin-A (DZNep) is an interesting agent that blocks EZH2. DZNep is a promising tool for cancer therapy. It induces apoptosis of cancer cells but not of their normal counterparts: epithelial cells and fibroblasts [9] or more importantly hematopoietic cells [10]. The action of DZNep is due to its inhibition of S-adenosylhomocysteine hydrolase, which disrupts methionine metabolism and results in methyltrasferases inhibition [3]. Similarly to EZH2 knockdown, DZNep reverts epithelial-to-mesenchymal transition (EMT), and prevents tumor progression, making it a highly promising anti-tumour agent [6]. A key feature of epigenetic alterations in malignancy is their relative plasticity, unlike genetic mutations to DNA sequence that are definitive [4]. Hereafter, there have been increasing numbers of reports showing the impressive anticancer effects of DZNep as a new epigenetic compound in a variety of cancer models both *in vitro* and *in vivo* [11–16]. The mechanisms and effects of DZNep have been studied in several solid tumors and acute myeloid leukemia, less is known about the potential of this compound for T-cell ALL [8].

Daunoblastine, a nonselective class I anthracycline, acts by binding to DNA-associated enzymes and intercalates the base pairs of the DNA’s double helix. Although daunoblastine has been used as an anti-leukemic agent for decades, its success is often dependent on combination with other drugs [17].

In the present study, we examined the expression levels of EZH2, EED and SUZ12 in samples of T cells ALL. Moreover, we evaluated the effects of DZNep alone or in combination with Daunoblastine on established T cell Jurkat line.

## Methods

Lymphoblastic leukemia cells were collected from pediatric patients diagnosed and treated for T-cell Acute Lymphoblastic Leukemia (T-ALL) at the Pediatric Oncology Unit of Second University of Naples and isolated from bone marrow at diagnosis with density gradient centrifugation Histopaque-1077 (1.077 g/ml; Sigma-Aldrich).

The study was approved by the Ethical Committee of the Azienda Universitaria Policlinico of the Second University of Naples (n. 94 on 21 January 2014) in compliance with the Helsinki Declaration. The informed consent for the participation to the study was approved and signed by the parents of the children.

### Protein extraction and western blot analysis

Protein extraction was performed on ice for 30 min using lyses-buffer with protease-inhibitors. Total protein concentration was determined using Bradford assay (Bio-Rad). 30 μg of total protein was run on 10 % polyacrylammide gel and blotted onto PVDF membrane (Millipore, Marlborough, MA). Immunoblotting was performed using primary antibodies against EZH2 (C-1), EED (H-300), SUZ12 (D-10) Bcl2 (C-2) (Santacruz Biotechnology, INC). Primary antibodies AKT, pAKT, ERK, pERK were obtained from Cell Signaling. All secondary antibodies were obtained from Santa Cruz Biotecnology. All antibodies were used in accordance with manifacturer’s instructions. Bands were visualized using a chemiluminescent system (ECL-Amersham). The intensity of each band was determined using a CCD camera and Quantity One 1-D analysis software (Biorad Laboratories). Results were normalized against the level of β-tubulin (Santa Cruz Biotechnologies) expression in each sample. It was obtained a range of expression of the bands from 0 to 175 %, with a median value of 60 %. Therefore, we have selected intensity values higher than 60 % in order to consider the expression of the different proteins as high. Values of the intensities associated to the specific bands of the different proteins lower or equal to 60 % were considered as low expression.

### RNA extraction and quantitative real-time PCR

Total RNA was extracted from cell cultures using TRI REAGENT (Molecular Research Center Inc., OH, USA) according to the manufacturer’s protocol. RNA from bone marrow at diagnosis was extracted with RNeasy FFPE kit (Invitrogen). The reactions were run on an ABI PRISM®7900HT Sequence Detection System; the cycling conditions were 10 min at 95 °C followed by 40 cycles of 15 s at 94 °C and 1 min at 68 °C. In the first step, we determined the stability of a control gene (β-actin) for the normalization of the real-time PCR products. Specific primers for human EZH2, SUZ12 and EED were designed (Table 1). Assays were performed in triplicate. We used the 2^-ΔΔCt^ method to analyze the data obtained.

**Table 1:** Primer sequences for quantitative real time-polymerase chain reaction

Gene	Sense	Antisense
EZH2	5′cgcttttctgtaggcgatgt 3′	5′tgggtgttgcatgaaaagaa 3′
SUZ12	5′gggagactattcttgatgggaag 3′	5′actgcaacgtaggtccctga 3′
EED	5′gaaattccatccaagagatcca 3′	5′tggatattccataatcgtaaagca3′
β-actin	5′gcgagaagatgacccagatc 3′	5′ggatagcacagcctggatag 3′

### Cell culture

Human T cell leukemia, Jurkat cell lines, obtained from the German Collection of Microorganisms and Cell Cultures (DMSZ) were grown in RPMI media supplemented with heat inactivated 10 % FBS and 1 % Penicillin/Streptomycin in a humidified atmosphere of 95 % air/5 % CO_2_ at 37 °C.

### Chemical reagents

DZNep was purchased from Sigma-Aldrich, dissolved in distilled water and stored as frozen aliquots at −20 °C. Daunoblastine was obtained from Pfizer Pharma and diluted in physiological saline solution.

### Cell viability assay

Cell viability was analyzed by MTT [3-(4, 5-dimethylthiazol-2-yl)-2, 5-diphenyl tetrazolium bromide] assay. Cells were seeded in 96-well plates at the density of 1 × 10^3^ cells/well in a final volume of 100 μL and then incubated at 37 °C in a humidified atmosphere. Cells were treated with DZNep at a range of concentrations between 0.15 and 24 μM/ml and with Daunoblastine at a range of concentrations between 2.5 to 150 ng/ml, in either single or combined treatments. Cells were exposed to a solution of 10 % MTT to 48 h and 72 h from the treatments, for 4 h at 37 °C to form formazan crystals by reacting with metabolically active cells. The formazan crystals were solubilized in a 1 N isopropanol/HCL 10 % solution at 37 °C for 30 min. The absorbance was determined at 595 nm [18]. Cell viability was determined by the formula: Cell viability (%) ‐ (absorbance of the treated wells ‐ absorbance of the blank control wells)/(absorbance of the negative control wells ‐ absorbance of the blank control wells) × 100 %. We have calculated 50 % growth inhibition index (IC 50) for DZNep and Daunoblastine.

### Drug combination studies

This study allowed the evaluation of the synergistic inhibition of cell growth produced by DZNep and Daunoblastine alone and in combination in Jurkat. Cells were seeded in 12-multi-weel plates at the density of 1 × 10^6^ cells/ml medium. After 24 h at 37 °C with 5 % CO_2_ in a humidified atmosphere, the cells were treated at the drugs range of concentrations described above. Drug combination studies were based on concentration-effect curves generated as a plot of the fraction of unaffected (surviving) cells versus drug concentration after 48 h of treatment. To explore the relative contribute of each agent to the synergism, the combination index (CI) for DZNep and Daunoblastine was obtained with software CalcuSyn. A CI of less than, equal to, and more than 1 indicates synergy, additivity, and antagonism, respectively as previously described [19].

### Cell cycle analysis

Jurkat cell line were seeded at the density of 1 x 10^6^ and treated with DZNep and Daunoblastine in humidified atmosphere at 37 °C for 48 h. After incubation, cells were washed in PBS, resuspended and directly stained in a Propidium Iodide (PI) solution for 30 min at 4 °C in the dark. Flow cytometry analysis was performed using FACScan (Becton Dickinson, San Jose, CA). To evaluate cell cycle, PI fluorescence was collected as FL2 (linear scale) by the ModFIT software (Becton Dickinson). For the evaluation of intracellular DNA content, at least 20,000 events for each point were analyzed in at least three different experiments giving a S.D. less than 5 %.

### Evaluation of apoptosis by DNA-flow cytometry

Apoptotis was analyzed by Annexin-V kit (MedSystems Diagnostics, Vienna, Austria). During the early stages of apoptosis, phosphatidylserine residues translocated from the inner to the outer leaflet of the plasma membrane binding Annexin-V-FITC. Jurkat cell line was treated with DZNep and/or Daunoblastine, for 48 h. After treatment, cells were collected and incubated with Annexin-V-FITC in a binding buffer (provided by the manufacturer) for 30 min at 4 °C. Analysis of apoptotic cells was performed by flow cytometry (FACS Aria III, Becton Dickinson). For each sample, 10x10^4^ events were acquired. Analysis was carried out by triplicate determination on at least three different experiments.

### AKT kinase assay

Cells were cultured and treated with DZNep and/or Daunoblastine and lysed as previously described [20]. Then, 20 μl of IgG1 anti-Akt monoclonal antibody agarose beads immobilized (Cell Signaling Technology) was added to 1 mg of cell lysate and the mixture was incubated at 4 °C for the night in gentle agitation. The resulting immunoprecipitates were then incubated for 30 min at 30 °C with 1 mg GSK-3 fusion protein (Cell Signaling Technology) in the presence of 200 mM ATP and Kinase Buffer (25 mM Tris, pH 7.5, 5 mM b-glycerophosphate, 2 mM dithiotreitol, 0.1 mM sodium orthovanadate, 10 mM MgCl2). The reaction was terminated with the addition of 20 μl 2 × SDS sample buffer. The supernatants were boiled for 5 min and electrophoresed by 12 % SDS‐PAGE and the protein electro-transferred on a nitrocellulose film. Phosphorylation of GSK-3 was detected using as probe an anti-Phospho-GSK-3a/b (Ser21/9) rabbit polyclonal antibody (diluted 1:1000) and then with a secondary anti-rabbit HRP-conjugated monoclonal antibody, (diluted 1:2000). The film was washed with TBS 13–0.05 % Tween 20 buffer and the specific reactivity was detected by chemiluminescence technique (Amersham) according to the manufacturer’s instructions (Cell Signaling Technology).

### Statistical analysis

PRC2 proteins and categories of different prognostic factors (gender, age, WBC count, relapse and prednisone response) was analyzed by chi-square test.

Then, the patients’ dichotomous variables were assessed according to their influence on relapse and disease-free survival (DFS). DFS estimated at 5 years by the Kaplan‐Meier analysis, was assessed using a log-rank test. In the DFS analysis, relapse and death in remission as a result of any cause were considered treatment failures. DFS was calculated for all patients that obtained complete remission from the date of remission to relapse. Results were expressed as probability (percent) and 95 % confidence intervals (CI). The hazard ratio (HR; with a 95 % CI) for PRC2 subunits was estimated by the Cox regression analysis and categorized according to relapse risk prediction with the common prognostic factors. Data are given as mean ± SD. Differences were assessed by *t* test and p values < 0.05 were considered statistically significant.

## Results

### Main clinical characteristics

Twenty patients (15 males and 5 females) were included in this study. The median age of the patients at diagnosis was 120 months. The whole group was enrolled in AIEOP-BFM treatment protocols. Morphologically the patients were stratified as L1 (4 cases) and L2 (16 cases), according to French American British (FAB) classification. At presentation, fourteen patients (77.8 %) had white blood cells (WBC) higher than 20,000/mmc and six (22.2 %) a lower count (Table 2). They were sub-divided into three risk categories, according to their prognostic parameters: standard risk (SR) if a white cell count below 20,000/mmc, DNA index between 1.16 and 1.60, good response to prednisone treatment (blasts reduction in peripheral blood <1,000/mmc after 7 days treatment with steroids), no translocation t (9;22) or t (4;11), complete remission at the conclusion of induction therapy; high risk (HR) if negative response to prednisone, evidence of translocation t(9;22) o t(4;11), not in remission at the conclusion of induction therapy, and Intermediate risk (IR) whose characteristics did not fall into either of the previous two categories. According to these features our patients were sub-classified as SR for 3 patients (15 %) and HR for 17 patients (85 %). Clinical, pathological data and evolution of the 20 patients are listed in Table 2. Overall, with a median follow-up of 31 months (95 % C.I 14.2–59.9), 11 patients (55 %) were alive and in continuous complete remission. Nine patients (45 %) relapsed.

**Table 2:** Clinical characteristics and pathologic data of 20 patients enrolled in the study

Clinical characteristics	Number of patients (%)
Gender	
Male	15 (75)
Female	5 (25)
WBC	
≤20.000/mm^3^	6 (30)
>20.000/mm^3^	14 (70)
FAB phenotype	
L1	4 (20)
L2	16 (80)
Disease Status	
Relapsed	9 (45)
No relapsed	11 (55)
Risk	
Standard	3 (15)
High	17 (85)
PDN	
GR	7 (35)
PR	13 (65)

*WBC* White Blood Cell, *PDN* Prednisone, *GR* Good Responders, *PR* Poor Responders

### PRC2 expression in T-ALL primary patients

**Fig. 1:**
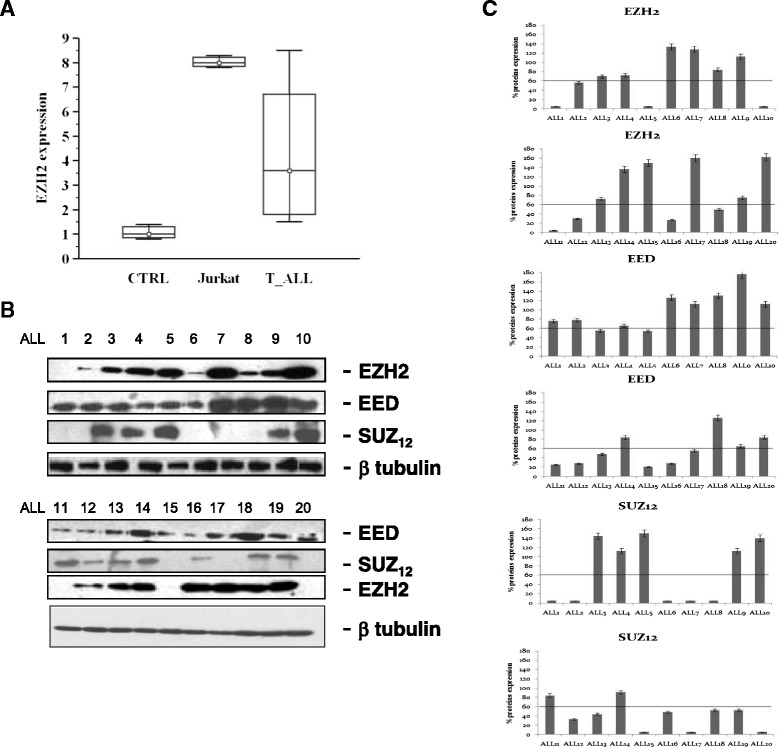
EZH2 Expression (**a**) EZH2 expression evaluated through real time PCR in sample control (CTRL), Jurkat and T-ALL samples. PRC2 expression evaluated through immunoblotting (**b**) in T-ALL samples enrolled in the study and (**c**) representation of the densities of the bands corresponding to EZH2, SUZ12 and EED expressed as percentage of expression after laser scanning

We have studied PRC2 expression and analysed the expression of mRNA in primary samples at diagnosis and on Jurkat cell line, by real time PCR based on RNA sample availability. EZH2 was markedly expressed like SUZ12 and EED (Fig. 1a); in details, the analysis showed from 0.5 to 7-fold higher expression of EZH2 compared to the control samples. We have also quantified protein expression by western blotting through the evaluation of the  densities of the corresponding bands after laser scanning and representation as percentage. We have calculated the cut off values and we considered “low” expression values less than or equal to 60 % and “high” expression higher than 60. EZH2 was expressed in 16/20 (75 %) samples at different extents: 4 samples (25 %) showed low expression levels, whereas 12 samples (75 %) had high levels. SUZ12 was expressed in 12 (60 %) samples; 5 (41.6 %) showed low levels and 7 (58.3 %) high expression. EED was expressed in all samples; 8 (40 %) had low expression and 12 (60 %) high expression (Fig. 1b-c). Moreover, Jurkat cell line expressed EZH2 and EED proteins only. When we compared EZH2, SUZ12 and EED expressions with clinical and biological characteristics of patients, we did not found any significant correlation (p > 0.05, data not shown).

### EZH2, SUZ12 and EED expression and correlation with clinical outcome

**Fig. 2:**
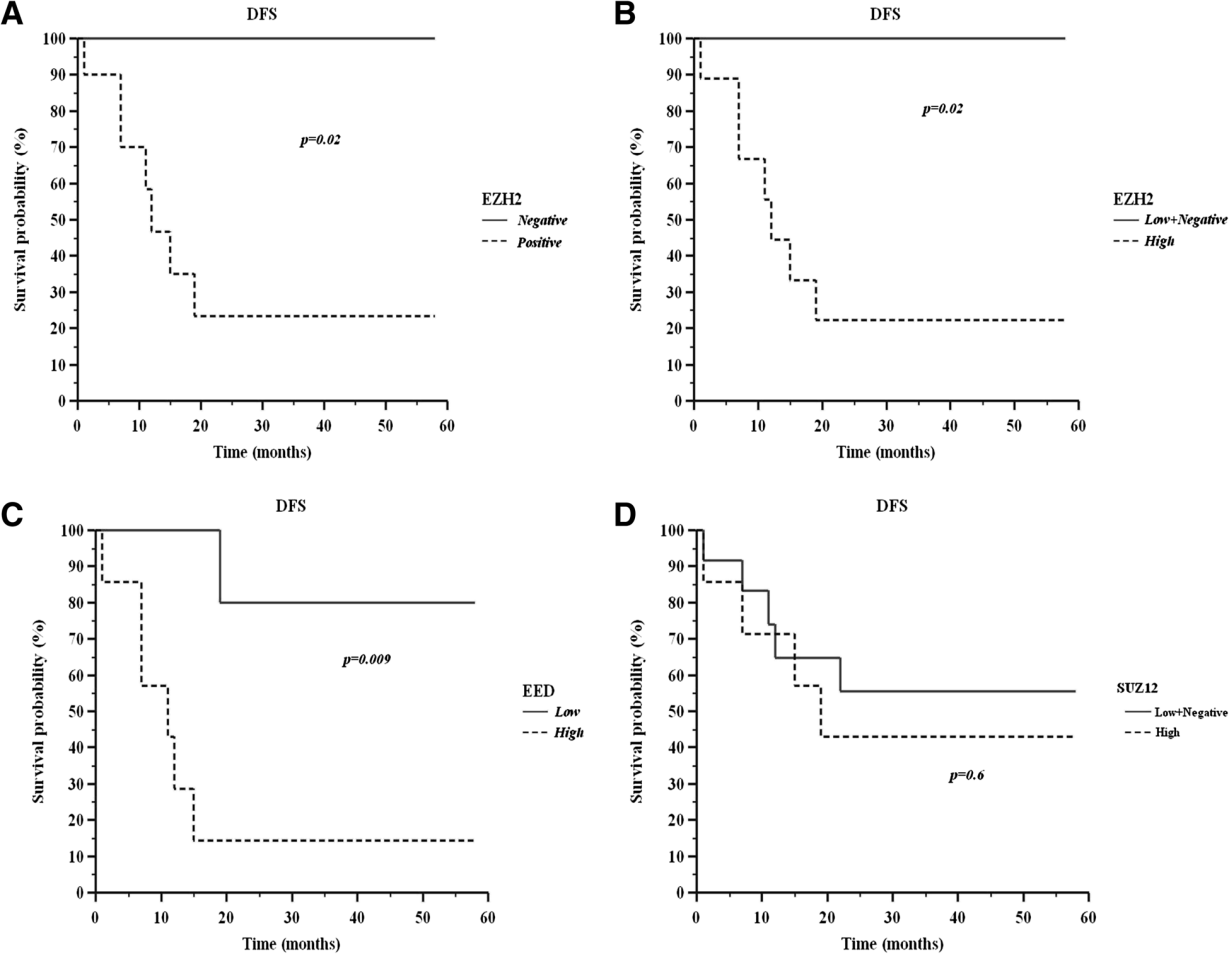
Kaplan–Meier analysis. Disease-free survival (DFS) and EZH2 expression (**a**, **b** ), EED expression (**c**) and SUZ12 expression (**d**)

Estimated 5-year DFS by the Kaplan‐Meier analysis shows that T-ALL expressing EZH2 had a lower probability of DFS compared to T-ALL negative cases for EZH2 (23 % vs 100 %) (log-rank test, p = 0.02, Fig. 2a). These data were confirmed when negative and low expression samples were grouped vs high expression (22 % vs 100 %) (log-rank test, p = 0.02, Fig. 2b). Moreover, Kaplan‐Meier analysis shows that patients with high EED expression had a lower probability of DFS vs low expression patients (14 % vs 86 %) (log-rank test, p = 0.009, Fig. 2c). No significant differences in DFS rate were found between patients stratified according to SUZ12 expression (43 % vs 57 %) (log-rank test, p >0.05, Fig. 2d). The Cox model, containing gender, age, initial WBC count, PDN response, FAB phenotype and EZH2 expression suggests that EZH2 is not an independent prognostic factor (p > 0.05).

### DZNep and Daunoblastine effects on Jurkat proliferation

**Fig. 3:**
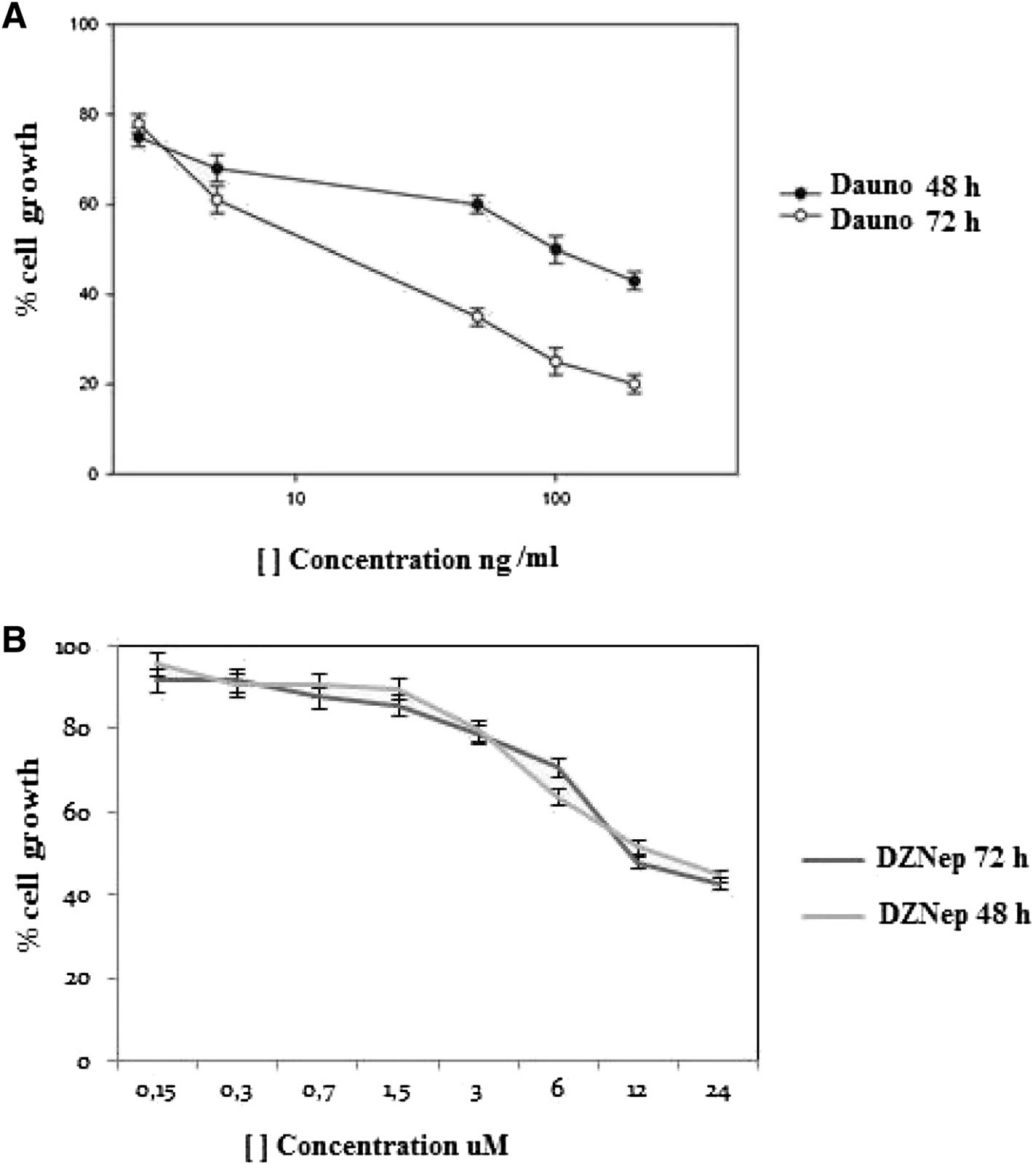
DZNep and Daunoblastine in Jurkat cell growth. Dose-dependent inhibitory effect of Daunoblastine (**a**) and DZNep (**b**) on Jurkat cells treated with different concentrations of DZNep or Daunoblastine at 48 and 72 h as described in Materials and Methods section. Percentage of cell growth induced by different concentrations of agents was evaluated with MTT assay. Each point is the mean of three different evaluations performed in at least three different experiments

Jurkat cell line is frequently used for delineating the mechanism of differential susceptibility of neoplasm for chemotherapy or radiation, which also emphasizes the significance of the line as a model for studies on T-acute lymphoblastic leukemia. DZNep and Daunoblastine reduced Jurkat number in a dose-dependent manner after 48 and 72 h from the beginning of incubation. DZNep induced 50 % growth inhibition (IC_50_) at 48 and 72 h with a concentration of 12 μM/ml, respectively, while Daunoblastine induced a less pronounced growth inhibition reaching the IC_50_ after 72 h at a concentration of 12 ng/ml. Moreover, only Daunoblastine was able to induce a time-dependent reduction of cell growth while DZNep reached a plateau after 48 h. (Fig. 3a-b)

### DZNep synergizes with Daunoblastine in Jurkat cell growth inhibition

**Fig. 4:**
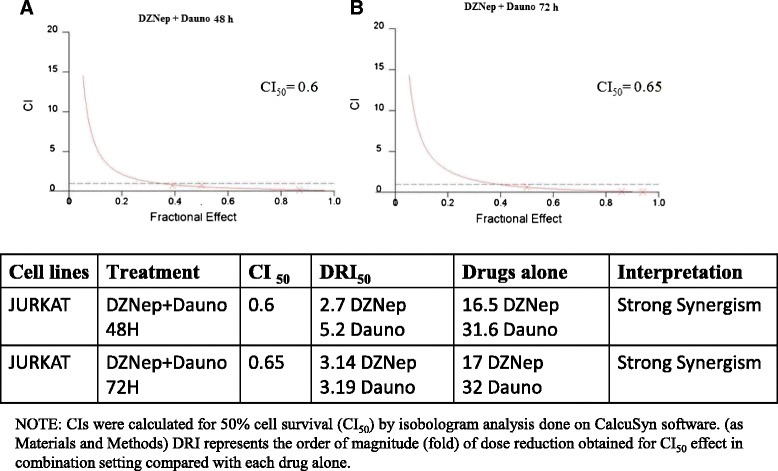
DZNep with Daunoblastine in Jurkat cell growth inhibition. DZNep and Daunoblastine have a synergistic effect on Jurkat cell growth inhibition. We have evaluated the growth inhibition induced by different concentrations of DZNep and Daunoblastine at 48 (**a**) and 72 h (**b**). We have performed these experiments with MTT assay and the resulting data were elaborated with the dedicated software Calcusyn as described in “Materials and Methods” section. In the figure, it is shown the isobologram analysis of the effects on growth inhibition of DZNep and Daunoblastine combinations, used (25:50) concentrations. CI, combination index. Each point is the mean of at least four different replicate experiments

We have evaluated the growth inhibition induced by DZNep in combination with Daunoblastine using data elaborated with the dedicated software Calcusyn (by Chou and Talalay, see also Materials and Methods section). The experimental CI_50s_ (combination index calculated for 50 % cell survival by isobologram analysis) values reveal that the DZNep/Daunoblastine combination was synergistic when both agents were used at (25:50 = Daunoblastine:DZNep) concentrations, in fact the CI_50s_ were 0.6 at 48 h and 0.65 at 72 h (Fig. 4a-b).

These results suggested that the activity of a combination based on the use of an EZH2 inhibitor (DZNep) with a conventional chemotherapy agent (already used in high risk ALL at the clinical setting) could be useful in EZH2 over-expressing cells.

### Cell cycle analysis after DZNep in combination with Daunoblastine treatment

**Fig. 5:**
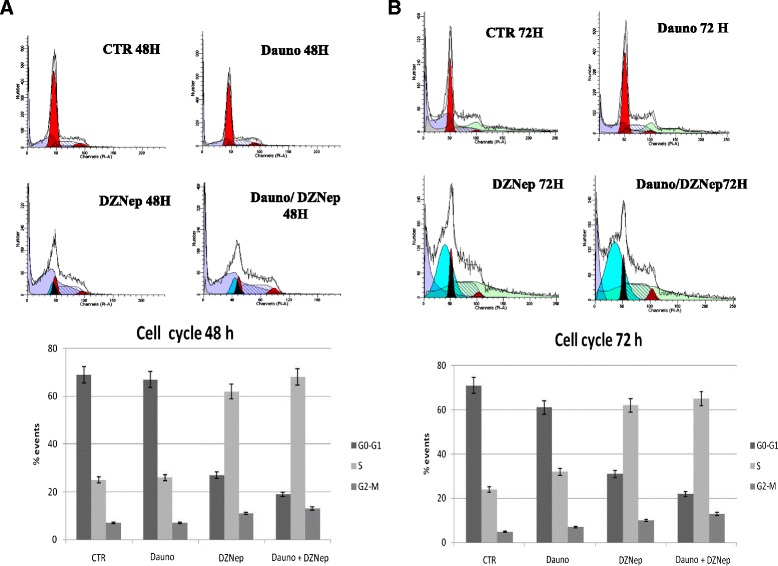
Effect of the DZNep and Daunoblastine treatment on cell cycle. We have evaluated the distribution of Jurkat cells at 48 h (**a**) and 72 h (**b**) in the different phases of the cell cycle, after the treatments with DZNep and/or Daunoblastine as previously described. This experiment was performed by FACS analysis after PI staining as described in Materials and Methods section

We determined the effects of DZNep or Daunoblastine alone and DZNep/Daunoblastine treatment on Jurkat cell cycle. As demonstrated in Fig. 5, at 48 h, DZNep treatment increased the percentage of cells in S-phase from 25 to 60 %; DZNep used in combination with Daunoblastine determined an additional increase of cells in S-Phase reaching an about 70 %. At the same time, cells in G_0/1_ phase decreased in a statistically significant manner (p < 0.001). These results on cell-cycle distribution were confirmed at 72 h (p <0.001).

### The synergistic effect of the DZNep/Daunoblastine combination on growth inhibition is paralleled by the apoptosis induction

**Fig. 6:**
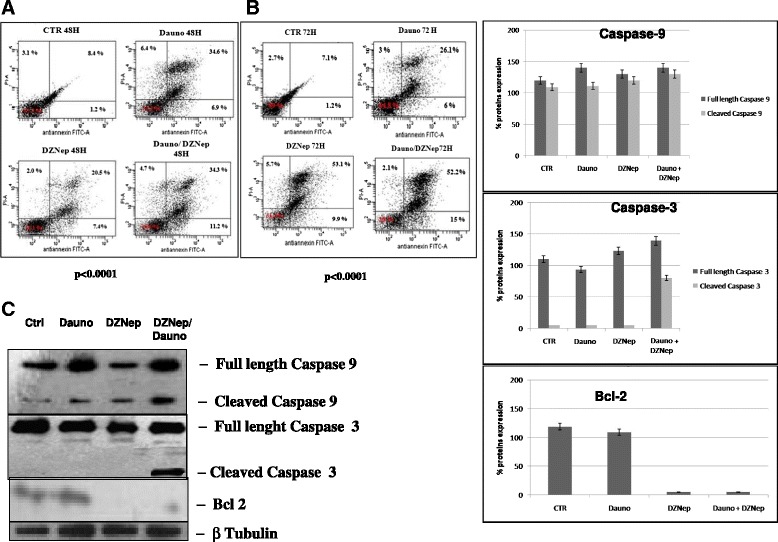
(**a**). The effect of DZNep/Daunoblastine combination on apoptosis. FACS analysis after double labelling of  Jurkat cells  with PI and Annexin V. Jurkat were treated with DZNep and Daunoblastine alone and in combination, compared to the control. The experiments were performed at least three times and the results were always similar. Insets, % of positive cells. UL = Upper Left (necrosis); UR = Upper Right (late apoptosis); LL = Lower Left (viable); LR = Lower Right (early apoptosis). Untreated cells, CTR; Dauno added for 48 h, DZNep 48 h; DZNep + Dauno added for 48 h. (**a**). The figure is representative of three different experiments that always gave similar results. The table shows the percentage of cells in the different quadrants. (**b**). FACS analysis after double labelling Jurkat with PI and Annexin V. Jurkat were treated with DZNep and Daunoblastine alone and in combination, compared to the control at 72 h. (**c**). Cells were then processed for the determination of the expression of caspase 3, caspase 9 and Bcl2 evaluated after blotting with specific antibodies described in Materials and Methods section. The house-keeping protein beta-tubulin was used as loading control. CTR, untreated; Dauno, DZNep and Dauno/DZNep treated cells as described before. Each point is representative of three different evaluations performed in at least three different experiments. Scan of the bands associated with caspase 3, caspase 9 and Bcl2 expression in Jurkat cells, normalized with the house-keeping protein, was performed with a dedicated software and the intensities of the bands were expressed as percentage protein expression (%, mean of three different experiments). Bars, s.e.’s

After the treatment with DZNep and Daunoblastine, used at IC_50_ concentrations as described above, we have evaluated the induction of apoptosis on Jurkat by FACS analysis, after staining with Annexin V-FITC and PI, as described above. As shown in Fig. 6, we found an increase of apoptotic cells treated with the two agents in combination, compared to untreated cells or cells treated with the single agents. In details, we found that the treatment with DZNep for 48 h induced apoptosis in about 41.5 % (34.6 % late and 6.9 % early) of Jurkat cell population, while the treatment with Daunoblastine for 48 h induced apoptosis in about 27.9 % (20.5 % late and 7.4 % early) (p < 0.0001) of Jurkat population compared to 9.6 % of untreated cells. On the other hand, when the cells were treated with DZNep + Daunoblastine, apoptosis was recorded in about 45.5 % (34.3 % late and 11.2 % early) of Jurkat cell population (p < 0.0001) (Fig. 6a).

It is noteworthy that DZNep treatment for 72 h induced apoptosis in 63 % (53.1 % late and 9.9 % early), Daunoblastine treatment in 32.1 % (26.1 % late and 6 % early) and the combination in about 67 % (52.2 % late and 15 % early) of Jurkat (p < 0.0001) (Fig. 6b). On the basis of these findings, it can be assumed that the combination was able to induce strong apoptosis that was paralleled by the synergism on cell growth inhibition.

On the basis of these results, we have evaluated the effect of treatment with the agents alone or in combination on the mechanisms of apoptotic occurrence. The combination determined an increase of caspase-3 and caspase-9 cleaved isoforms, as a marker of their activity. Supporting the hypothesis of the activation in the apoptotic pathway, we observed a complete depletion of Bcl-2 in Jurkat cells treated with DZNep alone and in combination with Daunoblastine (Fig. 6c).

### DZNep treatment: EZH2 modulation in Jurkat

**Fig. 7:**
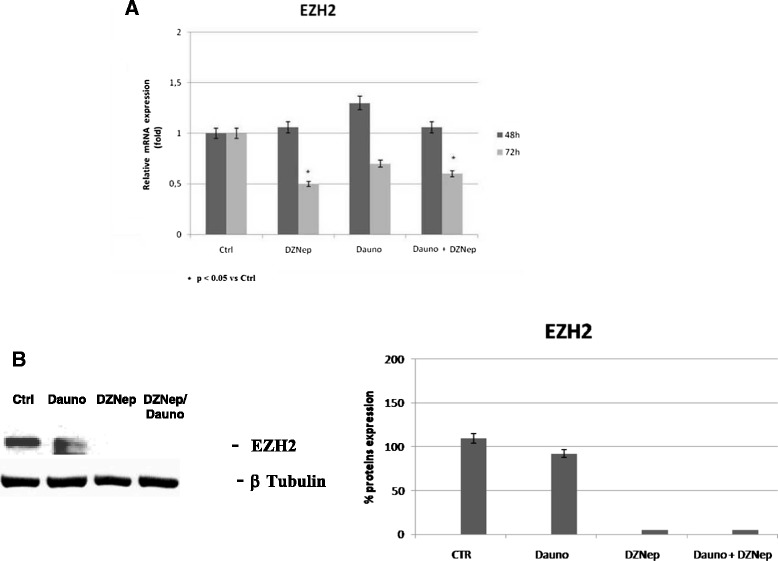
EZH2 modulation in Jurkat. (**a**) EZH2 expression evaluated through real time PCR in Jurkat at 48 and 72 h after treatment with Dauno, DZNep and Dauno + DZNep. (**b**) EZH2 expression evaluated through immunoblotting in Jurkat cell line treated at 48 h with Dauno, DZNep and Dauno + DZNep. The experiments were repeated three times and the values are the mean ± SD of the three independent experiments

We assessed EZH2 expression levels after DZNep and DZNep/Daunoblastine treatment in order to investigate upon the effects of the inhibitor in Jurkat cells. Quantitative RT-PCR analysis of EZH2 showed an about 50 % decreased expression in cells treated with the single agents or the combinations at 72 h (Fig. 7a). Moreover, we found an almost complete EZH2 protein depletion both in cells treated with DZNep and DZNep and Daunoblastine in combination at 48 h while an about 50 % EZH2 protein reduction was recorded in Daunoblastine-treated cells (Fig. 7b).

**Fig. 8:**
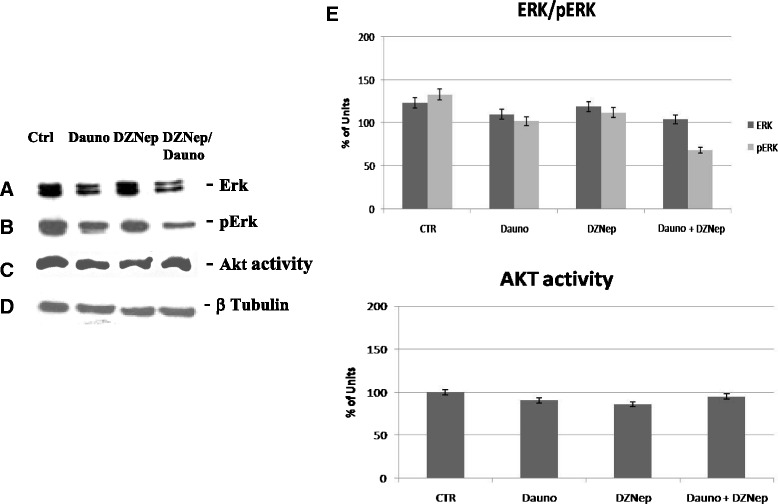
DZNep/Daunoblasatine combination on survival signal transduction. The cells were processed for the determination of the expression (**a**) and phosphorylation (**b**) of erk-1 and 2 evaluated after blotting with an anti-MAPK- and an anti-pMAPK-specific Mab, respectively, as described in ‘Materials and methods’. In the same experimental conditions, the activity (**c**) of Akt was also analysed with a Western blotting and an in-gel kinase assay, respectively, as described in ‘Materials and methods’. (**d**) Expression of the house-keeping protein beta-tubulin, used as loading control. The experiments were performed at least three different times and the results were always similar. CTR, untreated; Dauno; DZNep; Dauno/DZNep. (**e**) Scan of the bands associated with Erk 1/2 and Akt activity was performed with a dedicated software and the intensities of the bands were expressed as arbitrary units (%, mean of three different experiments). Bars, s.e.’s

### Effects of DZNep/Daunoblasatine combination on survival signal transduction

Finally, we studied the effects of the agents on both the expression and activity of two key molecules involved in the regulation of both cell proliferation and survival processes. In details, to elucidate the signaling pathways underlying the effects of DZNep on Jurkat cells, MAPK (Erk-1/phosphorylated Erk-1/2) and AKT were examined.

In Jurkat cells, DZNep alone did not induce modulation in Erk-1/2 expression and activity while Daunoblastine caused an about 50 % decrease of Erk-1/2 expression and phosphorylation if compared to untreated cells. The combined treatment strengthened the reduction of both activity and expression of Erk-1/2 (Figure 8 a, b and e).

We have found that DZNep alone and in combination did not induce significant changes in Akt expression. Moreover, we have evaluated the effect of the single agents and the combination on the modulation of Akt activity. In details, we found that neither the single agents nor the combination induced any significant changes on the activity of the survival Akt enzyme (Figure 8 c and e).

## Discussion

Stringent assessment of the relapse hazard in individual patients with ALL is an integral part of the modern approach to ALL therapy. Recently, the response to treatment is the most important prognostic factor in ALL. Indeed, many other variables have emerged as useful prognostic indicators. Moreover, the prognostic impact of age and, to a lesser extent, leukocyte count can be explained partly by their association with specific genetic abnormalities. It should be emphasized that primary genetic features do not entirely account for treatment outcome. For instance, up to 20 % of patients with favorable features as hyperdiploidy > 50 chromosomes or TEL-AML1 fusion continue to suffer recurrences for their leukemia [21]. Moreover, inactivation of the p53 gene through mutations or overexpression of HDM2 whose product can bind to p53 and induce its degradation, is associated with a poor prognosis in children with ALL. The independent prognostic importance of a patient’s gross early response to therapy has been recognized since the early 1980s. At the present, it is very important the measurement of minimal residual disease (MRD) by flow-cytometric detection of aberrant immunophenotypes or analysis by polymerase chain reaction (PCR) to afford a level of sensitivity and specificity that cannot be attained by traditional morphological assessment of treatment response [22].

In the last few decades, the survival in pediatric T-ALL patients has significantly improved, yet one patient in every four still encounters relapse and in most cases early. To date, few biomarkers have proved to be reliable in predicting T-ALL patient prognosis. The introduction of new factors that can lead to a more accurate patient risk stratification and the related treatment approach is expected to improve T-ALL outcome [22].

Recently, early T-cell precursor (ETP) ALL was identified as a T-ALL subgroup with a very poor outcome [23]. Moreover Zhang J et al. showed that ETP-ALL cases were characterized by activating mutations in genes regulating cytokine receptor and RAS signaling, inactivating lesions disrupting haematopoietic development, and histone modifying genes (EZH2, EED, SUZ12, SETD2 and EP300) [24].

EZH2 overexpression is implicated in tumorigenesis and correlates to poor prognosis in several tumour types [25].

Moreover, EZH2 is considered one of the most appealing epigenetic targets for therapy in human cancer but efficiency of how it regulates its target genes depends on a number of additional factors, which may differ from one tumor cell line to another, even of the same tissue [26]. Here we have shown an expression of EZH2 and EED in 20 samples of T cells ALL and our findings suggest that their over-expression gives a lower probability of DFS. Therefore, EZH2 expression might be taken in account for a more precise and reliable patient stratification in order to improve treatment selection.

Epigenetic therapy in patients with hematological malignancies has produced interesting responses, but few long-term survivors [3]. Daunoblastine has been used as an anti-leukemic agent for decades, its success is often dependent on combination with other drugs. An interesting epigenetic agent that has the potential to increase anti-leukemic action of Daunoblastine could be DZNep. DZNep was first studied to enlarge the antiviral drugs arsenal and was further shown to induce cancer cell death [27]. DZNep became a promising anti-tumor drug with a significant efficacy on various cell types and no evident toxicity *in vivo* [28]. Studies by Momparler et al. revealed that sequential treatment of 5-AZA-CdR followed by DZNep showed a synergistic interaction with respect to its antileukemic activity as shown by a loss of clonogenicity for both human myeloid and murine lymphoid leukemic cell lines [3]. In this study, we demonstrated for the first time that DZNep and Daunoblastine used in combination at 25:50 concentrations, were synergistic at the Calcusyn elaboration. This suggested the opportunity to use EZH2 inhibitor and Daunoblastine combination in the treatment of paediatric T-ALL. Indeed, we observed that the synergistic combinations produced also a strong pro-apoptotic effect on Jurkat cell line. In details, DZNep alone and in combination with Daunoblastine induced 63 % and 67 % of apoptosis at 72 h, respectively. It was shown that DZNep could inhibit the Jurkat cell proliferation activating probably check-points which cause cell cycle arrest at S phase, resulting in acceleration of apoptosis. In fact, in natural killer/T-cell lymphoma, EZH2 upregulated via Myc-mediated mRNA inhibition, directly activates cyclin D transcription and promotes cell proliferation independently from methyltransferase activity [29]. Despite the main function of EZH2 is gene silencing through the methylation of H3K27, several studies have shown that EZH2 acts as trascriptional activator in various types of cancer independently from H3K27me. For example in natural killer/T-cell lymphoma, EZH2 upregulation directly activates cyclin D transcription via Myc-mediated mRNA inhibition and promotes cell proliferation independently from methyltransferase activity [29]. In the same way, EZH2 upregulation found in Jurkat cell line likely activates cyclin D transcription explaining the effect of DZNep obtained in our experimental system. Our results revealed that the treatment with both agents changed the expression and activity of many important proteins including caspase-3, 9, Bcl-2, Erk and EZH2. Among these proteins, the down-regulation of Bcl-2 and up-regulation of cleaved Caspase-3 and Caspase-9 are on-line with apoptosis occurrence observed in cells treated with the combination. At the same time, the combination was also able to modulate Erk-mediated proliferation pathway. In details, we observed that Erk phosphorylation was significantly inhibited by the combined treatment. This observation raises the possibility that treatment regimen with DZNep + Daunoblastine might be used to treat T-ALL cell, particularly the subgroup at worse prognosis.

## Conclusion

Gene regulation by PRC2 complex is critical in tumorigenesis. Gene repression resulting from EZH2 activity is a key component of the epigenetic machinery with effects on both DNA and histone methylation. Our data on pediatric patients with T-ALL, confirm the involvement of EZH2 in the regulation of biological functions in T-ALL. Our study shows, for the first time, that reduction of EZH2 levels potentiates growth inhibition and apoptosis caused by Daunoblastine in Jurkat cell line. Pharmacological targeting of EZH2 might represent a potential feasible approach to be used as adjuvant treatment for making conventional therapy more effective in pediatric T-ALL.
